# An Electrostatically Self-Assembled Ternary Nanocomplex as a Non-Viral Vector for the Delivery of Plasmid DNA into Human Adipose-Derived Stem Cells

**DOI:** 10.3390/molecules21050572

**Published:** 2016-04-29

**Authors:** Sun-Hee Cho, Young-Woock Noh, Mi Young Cho, Yong Taik Lim

**Affiliations:** SKKU Advanced Institute of Nanotechnology (SAINT), School of Chemical Engineering, Sungkyunkwan University, Suwon 440-746, Korea; sunhc@skku.edu (S.-H.C.); woock@skku.edu (Y.-W.N.); fairylamp@naver.com (M.Y.C.)

**Keywords:** non-viral vector, ternary nanocomplex, self-assembly, hyaluronic acid, adipose derived stem cells

## Abstract

In this study, we developed electrostatically self-assembled ternary nanocomplexes as a safe and effective non-viral vector for the delivery of plasmid DNA (pDNA) into human adipose-derived stem cells (hASCs). Although polyethylenimine (PEI) polymers initially showed excellent performance as gene delivery carriers, their broad use has been limited by cytotoxicity resulting from their strong positive charge. To reduce the cytotoxicity, we utilized anionic hyaluronic acid (HA) as a corona layer material for pDNA/PEI binary nanocomplexes. HA was also introduced to increase the targeting efficiency of pDNA/PEI nanocomplexes because HA has can bind CD44 that is highly expressed on the surface of hASCs. We confirmed that the addition of HA changed the surface charge of pDNA/PEI nanocomplexes from positive to negative. The pDNA/PEI/HA ternary nanocomplexes showed high transfection efficiency and low cytotoxicity compared with commercially available products. When hASCs were pretreated with HA to passivate CD44, the transfection efficiency of pDNA/PEI/HA nanocomplexes was significantly reduced. These results suggest that HA that can act as a targeting ligand to CD44 contributed to the improved transfection of pDNA into hASCs. Our novel pDNA/PEI/HA nanocomplexes may be used as an effective non-viral pDNA delivery system for hASCs.

## 1. Introduction

**Figure 1:**
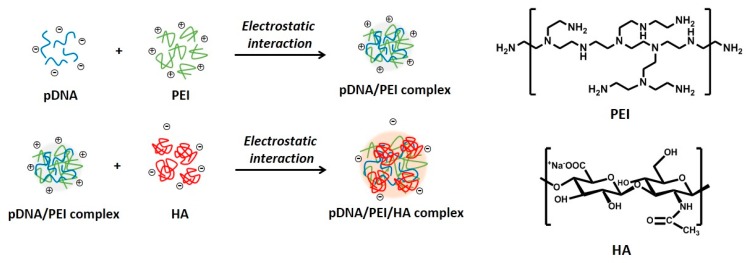
Schematic illustrations for the synthesis of electrostatically self-assembled pDNA/PEI/HA ternary nanocomplexes (pDNA (**blue line**): plasmid DNA, PEI (**green line**): polyethylenimine, HA (**red line**): hyaluronic acid).

Mesenchymal stem cells (MSCs) are a promising source for stem cell-based regenerative medicine because of their ability to differentiate into multiple cell types including bone, cartilage, fat, and muscle [1,2,3,4,5]. Thus, controlling MSC growth and differentiation by genetic modification represents a crucial strategy for improving the regenerative ability of MSCs in injured tissue. In this context, transferring target genes into MSCs by a variety of delivery methods is a powerful tool for controlling MSC function [6]. Current methods for introducing functional genes into MSCs involve vectors derived from viruses such as lentiviruses, retroviruses, and adenoviruses [6,7,8]. However, the clinical use of viral gene delivery vectors presents some problems, such as mutagenesis, pathogenic risk, and triggering a host immune response. In addition, limitations in exogenous DNA packaging capacity and large scale production remain, although viral vectors can efficiently deliver genes into cells [9]. Non-viral gene delivery vectors can overcome problems associated with viral methods, although non-viral gene delivery vectors also have some issues, such as high toxicity, low transfection efficiency, and biodegradability [9,10,11]. To increase cellular uptake of non-viral gene delivery vectors, previous studies have focused on the use of biocompatible polymers. Recently, ternary complexes have been developed using anionic biocompatible polymers such as alginic acid [12], heparin [13], and poly (γ-glutamic acid) [14], to reduce cytotoxicity and improve the transfection ability of non-viral gene delivery vectors. Hyaluronic acid (HA) is one such polymer that is negatively charged at physiologic pH values, and is a component of the extracellular matrix throughout the body. HA is also known to specifically bind the CD44 receptor. MSCs including bone marrow-derived and adipose-derived MSCs are generally known to have hyaladherins such as CD44, CD54, and CD168 present on their cell surfaces [15]. In previous studies, low molecular weight HA improved transfection efficiency of polyplexes by CD44 receptor-mediated uptake in human corneal epithelial cells [16]. In this study, we developed electrostatically self-assembled ternary nanocomplexes by incorporating branched polyethylenimine (PEI) as a plasmid DNA carrier and low molecular weight HA (4–8 kDa) not only to facilitate CD44 receptor-mediated uptake of ternary nanocomplexes into human adipose-derived MSCs (Figure 1), but also to reduce the toxicity of cationic PEI. After fabrication and characterization of our fabricated pDNA/PEI/HA ternary nanocomplexes, their transfection efficiency was investigated and compared with the performance of the commercial lipid-based agent X-tremeGENE.

## 2. Results and Discussion

### 2.1. Characterization of pDNA/PEI Binary and pDNA/PEI/HA Ternary Nanocomplexes

**Figure 2:**
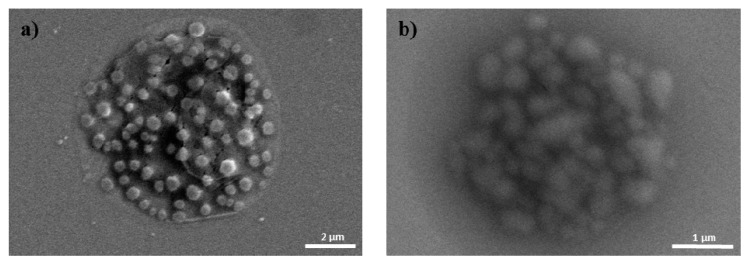
SEM images of (**a**) pDNA/PEI; and (**b**) pDNA/PEI/HA (1:8:20).

Due to the cationic properties of branched PEI (Mw = 25 kDa), this material has been intensively studied as a non-viral gene delivery vector both *in vitro* and *in vivo* [17]. PEI complexes with DNA via an electrostatic interaction, binds to the cell surface, and releases the encapsulated DNA into the cytoplasm by a proton sponge mechanism [18]. However, it is still not suitable for clinical application due to toxicity and low transfection efficiency [10]. In this study, we prepared ternary nanocomplexes of pDNA/PEI/HA by electrostatic self-assembly to overcome the problems of toxicity and low transfection efficiency. HA can be easily assembled with other materials via electrostatic interactions due to the presence of many carboxyl and hydroxyl groups (Figure 1). The previous studies showed that optimal transfection efficiency was observed at an N/P ratio of 8 in human MSCs [19]. So, we produced pDNA/PEI binary nanocomplexes by the self-assembly of negatively charged pDNA and positively charged PEI at N/P ratio of 8. For the ternary pDNA/PEI/HA nanocomplexes, the relative mixing ratio (*i.e.*, phosphate of pDNA: nitrogen of PEI: carboxylate of HA) was controlled as shown in Table 1. The sizes and zeta potentials of pDNA/PEI and pDNA/PEI/HA nanocomplexes were determined by dynamic light scattering (DLS), and the results are shown in Table 1. The size of pDNA/PEI (1:8) obtained was 626 ± 106 nm, and that of pDNA/PEI/HA was increased when HA was added as corona layer. The negatively charged HA may loosen the compact ionic pDNA/PEI nanocomplexes. The surface of the pDNA/PEI complexes showed a positive charge (+12.24 mV), while it was changed to a negative charge after the addition of HA depending on the amount of HA added (pDNA/PEI/HA (1:8:5): −7.49 mV, pDNA/PEI/HA (1:8:10): −12.40 mV, and pDNA/PEI/HA (1:8:20): −16.05 mV) due to HA’s carboxyl group (Table 1). When the morphology of pDNA/PEI and pDNA/PEI/HA nanocomplexes was observed by scanning electron microscopy (SEM), pDNA/PEI nanocomplexes showed particle-like complexes. Whereas, pDNA/PEI/HA nanocomplexes showed a more swollen and smooth surface (Figure 2). This observation makes sense because positively charged PEI binds strongly with negatively charged pDNA resulting in a tight and particle-like structure, and the addition of negatively charged HA would loosen this tight structure. The changes seen in surface charge and morphology between pDNA/PEI and pDNA/PEI/HA nanocomplexes strongly suggested that anionic HA was distributed as corona layer on the ternary nanocomplexes.

**Figure 3:**
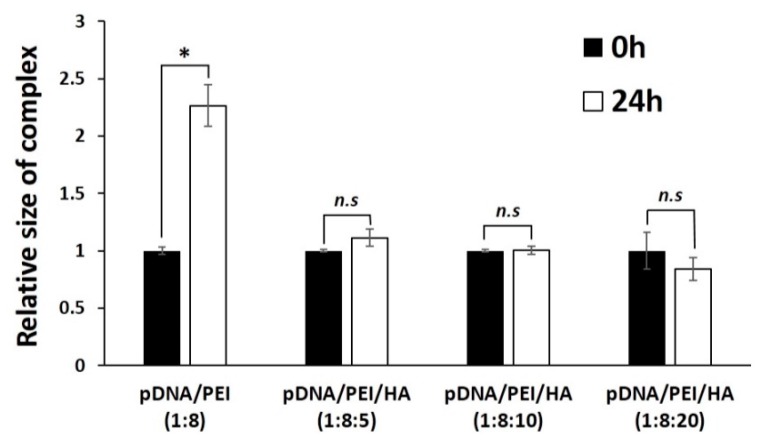
The size stability of each nanocomplex after incubation for 24 h. * *p* < 0.05, *n.s* = not significant.

**Figure 4:**
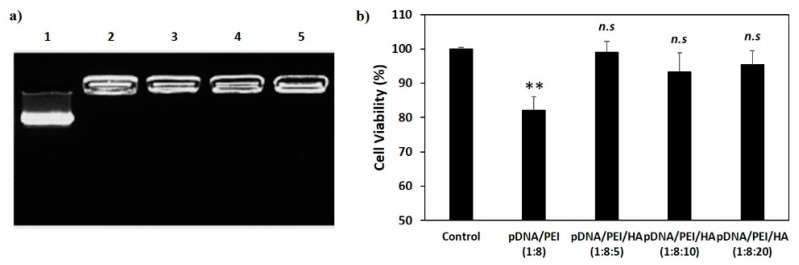
(**a**) Gel retardation assay of nanocomplexes. 1. Naked pDNA; 2. pDNA/PEI (1:8); 3. pDNA/PEI/HA (1:8:5); 4. pDNA/PEI/HA (1:8:10); and 5. pDNA/PEI/HA (1:8:20); (**b**) Viability of hASCs treated with each nanocomplex formulation as measured by MTS assay (*n* = 3). hASCs were incubated with various nanocomplexes for 24 h and cell viability was measured. ** *p* < 0.01 *vs.* control. *n.s* = not significant *vs.* control.

We measured the stability of pDNA/PEI and pDNA/PEI/HA complexes after incubation for 24 h. After incubation, the size of pDNA/PEI/HA complexes was unchanged, whereas the size of pDNA/PEI complexes was increased about 2.3-fold (Figure 3). These results suggested that the addition of HA also contributed to the stability of ionic nanocomplexes. To investigate the stability of pDNA encapsulation in pDNA/PEI/HA nanocomplexes prepared with different concentrations of HA, a gel retardation assay was carried out using gel electrophoresis. Figure 4 shows images of the gel retardation assay with pDNA/PEI and pDNA/PEI/HA nanocomplexes at various mixing ratios. Although a naked pDNA band was detected in the pDNA control lane, no such pDNA band was detected in any lane containing nanocomplexes fabricated at any mixing ratio as indicated in Table 1 (Figure 4a). Because the zeta potential value (−7.49 ± 0.44) of pDNA/PEI/HA (1:8:5) was closest to a neutral charge (*i.e.*, charge-conversion point from positive to negative) of all the ternary complexes examined, we assumed that the role of HA in loosening the tight complex structure was dominant at that composition resulting in a bigger size. The results of this experiment suggested that the pDNA was successfully encapsulated in pDNA/PEI and pDNA/PEI/HA nanocomplexes, and remained in a stable state even after the addition of HA.

### 2.2. Viability of hASCs Treated with pDNA/PEI Binary and pDNA/PEI/HA Ternary Nanocomplexes

The cytotoxicity of a gene delivery vector is one of the important factors to be considered before clinical application. However, highly cationic polymers and commercial gene transfection reagents are commonly known to be toxic [6,9]. To investigate possible cytotoxic effects of pDNA/PEI and pDNA/PEI/HA nanocomplexes, hASCs were treated with each nanocomplex formulation and cell viability was measured by MTS assay. As shown in Figure 4b, pDNA/PEI nanocomplexes were toxic to hASCs, but pDNA/PEI/HA nanocomplexes did not show any significant cytotoxicity.

### 2.3. Transfection Efficiency of pDNA/PEI Binary and pDNA/PEI/HA Ternary Nanocomplexes

**Figure 5:**
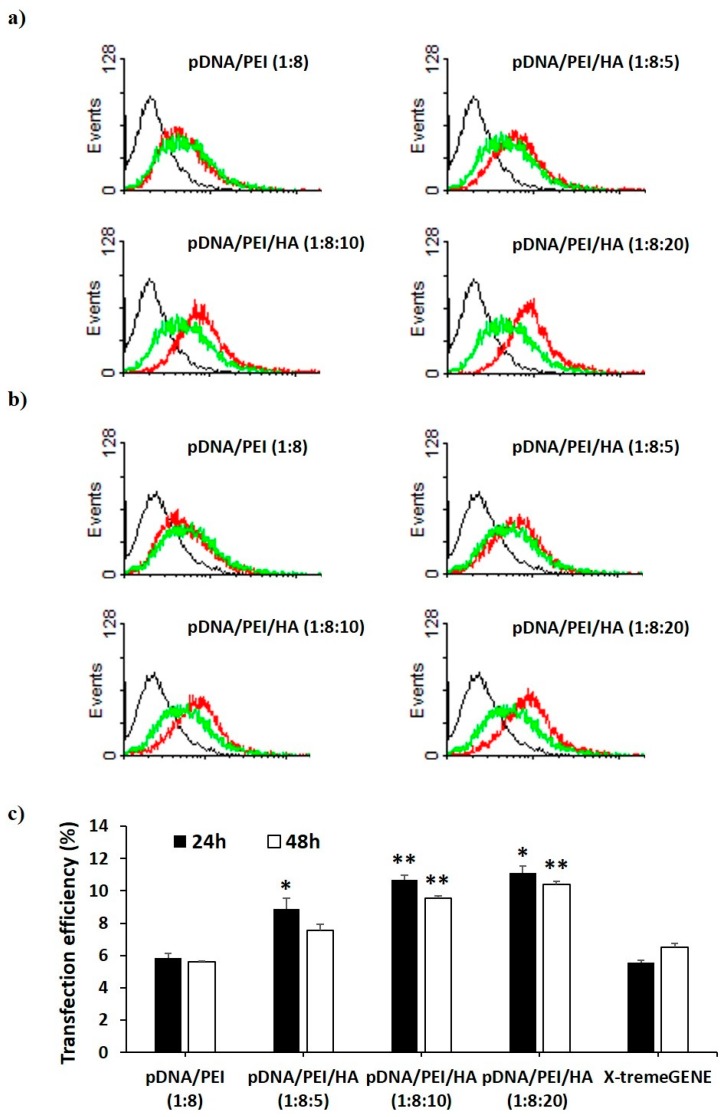
Flow cytometry profiles of non-transfected hASCs (**black**) and hASCs transfected with pCXLE-EGFP using each nanocomplex (**red**) and X-tremeGENE (**green**) (**a**) at 24 h and (**b**) 48 h after treatment. Black line = control, red line = complex, green line = X-tremeGENE; (**c**) Percentage of EGFP-expressing hASCs after transfection by each nanocomplex. Data are presented as means ± S.E. (*n* = 4), * *p* < 0.05, ** *p* < 0.01 *vs.* X-tremeGENE.

We investigated the transfection efficiency of pDNA/PEI and pDNA/PEI/HA nanocomplexes on hASCs *in vitro* through flow cytometry and fluorescence microscopy. We also compared the transfection efficiency of pDNA/PEI nanocomplexes (positive net charge) and pDNA/PEI/HA ternary nanocomplexes (negative net charge) with the commercial transfection product, X-tremeGENE. As a model pDNA, a plasmid containing enhanced green fluorescent protein (EGFP) was used. Flow cytometry experiments measured the percentage of GFP-positive cells to determine transfection efficiency. As shown in Figure 5, the transfection efficiency of pDNA/PEI binary nanocomplexes was about 6% which was similar to the efficiency of X-tremeGENE. Transfection efficiency was relatively increased in all pDNA/PEI/HA ternary nanocomplexes. Compared with transfection efficiency of pDNA/PEI or X-tremeGENE, 1.6- to 2.0-fold increases were observed for pDNA/PEI/HA ternary nanocomplexes (Figure 5). Some cytotoxicity and aggregation were observed when hASCs were treated with the positively charged pDNA/PEI nanocomplexes in serum-containing medium. This may be related to the low transfection efficiency. In contrast, the net negative surface charge of pDNA/PEI/HA nanocomplexes reduced the membrane destabilization of negatively charged hASCs. It should be also emphasized that HA binds CD44 which is highly expressed on the surface of hASCs. After binding CD44 on the cell surface, pDNA/PEI/HA nanocomplexes could enter the cells via receptor-mediated endocytosis. The expression of transfected EGFP pDNA in hASCs after transfection was measured by fluorescence image analysis. 

**Figure 6:**
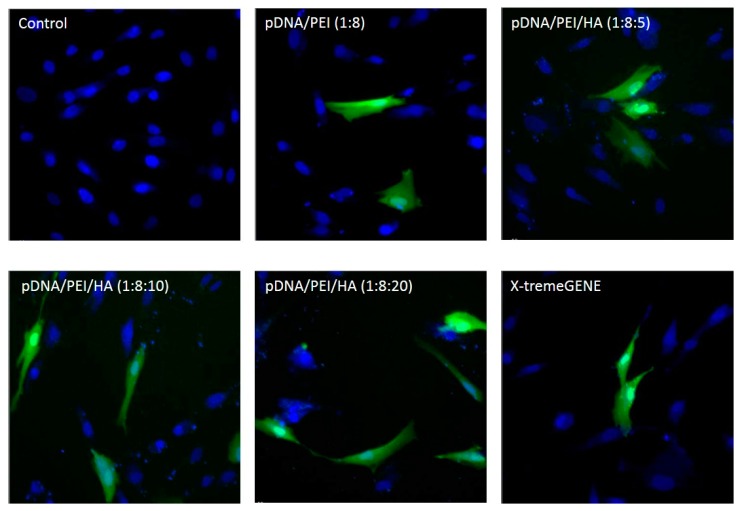
Fluorescence images of hASCs after transfection with each nanocomplex at 24 h. (Ex 490/20 and 360/40; Em 526/36 and 455/50).

As shown in Figure 6, EGFP expression was higher in hASCs when the plasmid gene was transfected using pDNA/PEI/HA ternary nanocomplexes compared to when pDNA/PEI or X-tremeGENE was used.

### 2.4. CD44 Targeting Effect of pDNA/PEI/HA Ternary Nanocomplexes

**Figure 7:**
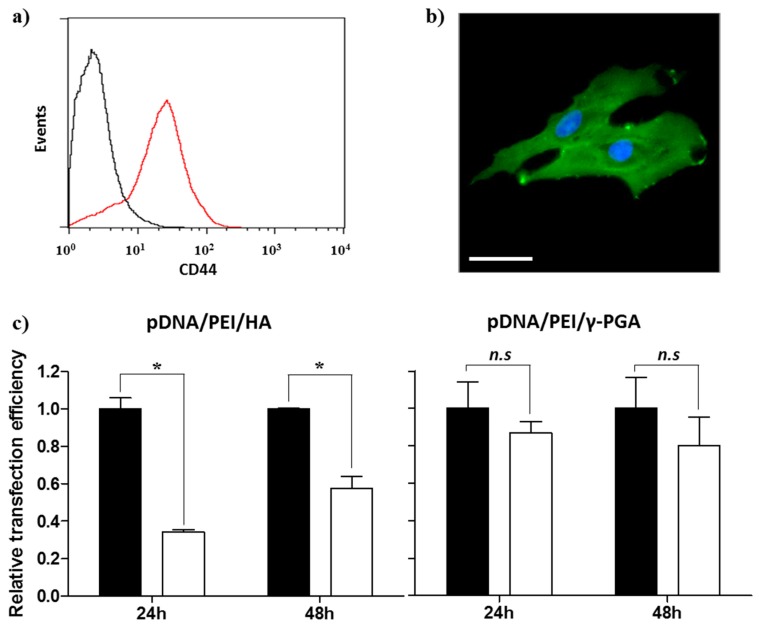
Expression of CD44 in hASCs evaluated by (**a**) flow cytometry and (**b**) fluorescence image. Scale bar = 40 µm. (**c**) Competition assay to investigate the effect of HA pretreatment on the transfection efficiency of pDNA/PEI/HA (left) and pDNA/PEI/γ-PGA (right) ternary nanocomplexes at 24 or 48 h. ■ = without HA pretreatment, □ = with HA pretreatment. Data are presented as means ± S.E. (*n* = 4), * *p* < 0.05, *n.s* = not significant *vs.* untreated control.

**Figure 8:**
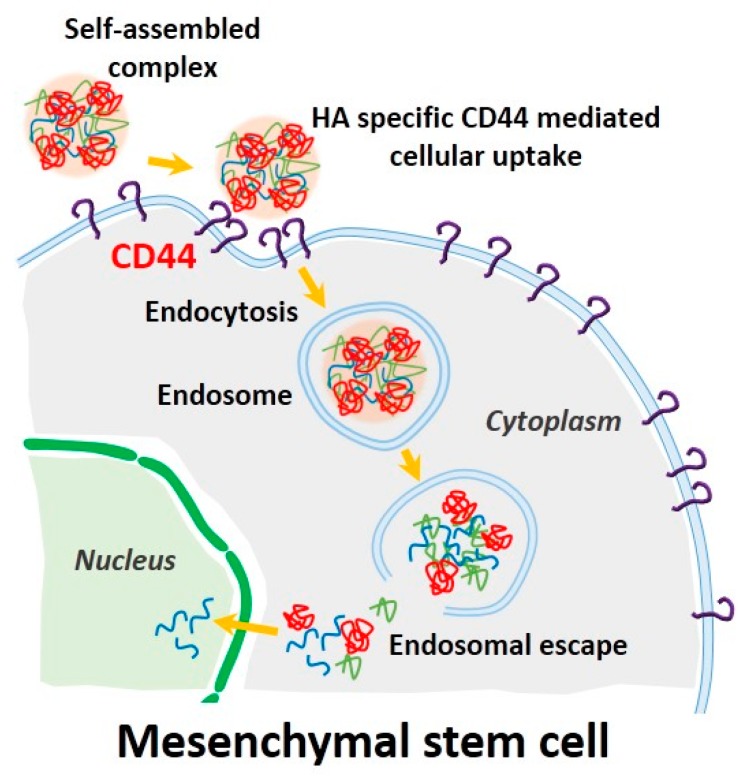
Schematic illustration of CD44-mediated endocytosis and delivery of pDNA into a stem cell using our self-assembled complexes. (Blue line: plasmid DNA, green line: PEI, red line: HA).

Prior to evaluating the CD44-targeting effect of pDNA/PEI/HA, we first confirmed the degree of CD44 receptor expression on the surface of hASCs membrane using an FITC-conjugated anti-CD44 antibody. Through flow cytometry and fluorescence microscopy images, we confirmed that CD44 was highly expressed on hASC surfaces (Figure 7a,b). To confirm that the pDNA/PEI/HA nanocomplexes were taken into hASCs by an HA-specific CD44 receptor-mediated pathway, hASCs were transfected with pDNA/PEI/HA ternary nanocomplexes after blocking CD44 by pretreatment with free HA. As shown in Figure 7c, transfection efficiency was significantly decreased when CD44 was blocked by free HA. As another control experiment, negatively charged poly (γ-glutamic acid) (γ-PGA) was used as a corona layer to form pDNA/PEI/γ-PGA ternary nanocomplexes [20]. However, the gene transfection efficiency of pDNA/PEI/γ-PGA ternary nanocomplexes showed little change after pretreatment with HA. These results strongly suggest that pDNA/PEI/HA complexes were taken into hASCs through CD44 receptor-mediated pathway. Through these experiments, we confirmed that HA can be utilized as a corona layer for pDNA/PEI binary nanocomplexes to increase the targeting these nanocomplexes to CD44 which is highly expressed on hASC surfaces, and to reduce toxicity (Figure 8).

## 3. Materials and Methods

### 3.1. Materials

Oligomeric hyaluronic acid (oligo-HA, Mw = 4-8 kDa) was purchased from Bioland (Cheonan, Korea). PEI (branched form, Mw = 25 kDa) was purchased from Sigma-Aldrich (St. Louis, MO, USA). The γ-PGA (salt form, Mw = ~50 kDa) was obtained from Bioleaders Corporation (Daejeon, Korea). MesenPRO RSTM Medium Kit and L-glutamine were purchased from Life Technologies (Carlsbad, CA, USA). X-tremeGENE HP DNA Transfection Reagent was purchased from Roche Diagnostics (Indianapolis, IN, USA). FITC rat anti-mouse CD44 antibody was purchased from BD Pharmingen (San Diego, CA, USA). All other reagents were molecular biology grade.

### 3.2. hASCs Culture

The hASCs (StemPro Human Adipose-Derived Stem Cells) were purchased from Life Technologies and cultured in 75-cm^2^ flasks using MesenPRO RSTM Medium Kit and L-glutamine according to the manufacturer’s instructions.

### 3.3. Preparation and Characterization of Ternary Nanocomplexes

Plasmid DNA (pDNA; pCXLE-EGFP, 10.9 kbp) from addgene (Cambridge, MA, USA) was amplified in *E. coli*, and then extracted and purified using a plasmid purification midi kit (Qiagen, Valencia, CA, USA). The pDNA concentration was determined by measuring UV absorbance of the sample at 260 nm. The pDNA/PEI/HA ternary complexes were prepared as shown in Figure 1. First, pDNA/PEI binary complexes with an N/P ratio of 8 were prepared by mixing 1 mL of pDNA (100 µg/mL) with 1 mL of PEI and incubating the mixture for 30 min at room temperature. Then, 1 mL of HA solution in MOPs buffer (varying concentrations) was added to obtain pDNA/PEI/HA ternary complexes with N/P/C ratios of 5, 10, and 20, followed by incubation for 30 min at room temperature. 

The size, size distribution, and surface charge of the complexes were measured via dynamic light scattering (DLS) (ELS-Z, Photal, Otsuka, Japan) within 1 h after complex formation. Morphology of the complexes was observed using a scanning electron microscope (SEM, JSM-7000F, JEOL Ltd. Tokyo, Japan). To prepare samples for SEM analysis, samples were diluted with deionized water and dropped onto silicon wafers. After that, samples were dried for 24 h at room temperature.

### 3.4. Gel Retardation Assay

pDNA binding capability of polymer in the complexes was determined by an agarose gel retardation assay. The complexes were prepared as described above, mixed with DNA loading buffer, and loaded into a 1.0% agarose gel in tris-acetate-EDTA (TAE) buffer containing 0.5 µg/mL ethidium bromide (Sigma-Aldrich). The samples were electrophoresed at 100 V for 30 min. The gel was visualized using a UVP M-20 Benchtop Transilluminator and BioDoc-It Imaging System (UVP, Upland, CA, USA).

### 3.5. Cell Viability Assay

The cytotoxicity of the complexes was evaluated using MTS-based CellTiter 96 AQueous Assay reagent (Promega, Madison, WI, USA) according to manufacturer’s instructions. Briefly hASCs were plated at a density 1 × 10^4^ cells per well in flat-bottomed 96-well plates (Corning Costar, Cambridge, MA, USA) and were incubated at 37 °C. After 24 h, the hASCs were incubated with 4 µL of pDNA/PEI binary complex or 6 µL of pDNA/PEI/HA ternary complexes for 24 or 48 h. For the MTS assay, 10 µL of MTS reagent was added into each well containing 100 µL of culture medium, and then the plates were incubated for 3 h at 37 °C. Absorbance of the wells was detected at 490 nm by a microplate reader (VersaMax, Molecular Devices, Sunnyvale, CA, USA).

### 3.6. Determining Transfection Efficiency by Flow Cytometry

For transfection, hASCs were seeded at a density 1 × 10^5^ cells per well in 6-well plates (Corning Costar) and incubated for 24 h at 37 °C. Then, the hASCs were incubated with 40 µL of pDNA/PEI binary complex or 60 µL of pDNA/PEI/HA ternary complexes containing 2 µg of pDNA for 24 h. For transfection using X-tremeGENE reagent, hASCs were incubated with 2 µg of pDNA and 8 µL of X-tremeGENE reagent. The pDNA and X-tremeGENE mixture was prepared according to the manufacturer’s protocol. When evaluating transfection efficiency at 48 h after complexes treatment, medium with samples was replaced with fresh medium containing the appropriate reagent at 24 h after samples treatment. After an additional 24 h of incubation, hASCs were harvested, washed with PBS twice, and suspended in 200 µL of 4% (*w*/*v*) paraformaldehyde solution for 20 min. The transfection efficiency of hASCs treated with each complex was expressed as the percentage of GFP-expressing cells measured by flow cytometry (MACSQuant analyzer, Miltenyi Biotec, Bergisch Gladbach, Germany). To confirm the CD44-blocking effect of HA pretreatment, hASCs were seeded at a density 1 × 10^5^ cells per well in 6-well plates with or without HA and were incubated for 24 h at 37 °C. Culture media were replaced with fresh media after 24 h. Next, the hASCs were incubated with 60 µL of pDNA/PEI/HA or pDNA/PEI/γ-PGA ternary complexes containing 2 µg of pDNA. After incubation for 24 h, hASCs were harvested to determine transfection efficiency.

### 3.7. Fluorescence Microscopy Imaging

To obtain the fluorescence images of hASCs after transfection with each sample, hASCs were incubated with complexes in a μ-slide 8-well chamber (Ibidi, Martinsried, Germany) at a density of 1 × 10^4^ cells per well for 24 h at 37 °C. The cells were then washed in cold PBS, fixed with 4 % (*w*/*v*) paraformaldehyde solution for 20 min at room temperature and stained with 2 µg/mL Hoechst 33342 (trihydrochloride, trihydrate, Life Technologies) in PBS for 15 min. Fluorescence images were obtained using a DeltaVision PD (Applied Precision Technologies, Issaquah, WA, USA, filters set to excitation: 490/20 and 360/40; emission: 526/36 and 455/50) (Omega Optical, Brattleboro, VT, USA).

## 4. Conclusions

In conclusion, we developed pDNA/PEI/HA ternary nanocomplexes as a promising non-viral vector for the delivery of pDNA into hASCs. The ternary nanocomplexes were easily fabricated by electrostatic interaction between components, and the complexes showed high transfection efficiency and low toxicity. Through a competition assay, we found that the pDNA/PEI/HA nanocomplexes were taken up by hASCs through an HA-specific CD44-mediated pathway, and the complexes could successfully deliver a target gene into hASCs. Therefore, biocompatible HA can be used to modify surfaces of cationic pDNA/PEI nanocomplexes not only to reduce toxicity, but also to bind CD44 and increase targeting efficiency to hASCs. Based on these findings, our novel pDNA/PEI/HA ternary nanocomplexes containing specific target pDNA are proposed for use in genetic modulation of stem cells as well as other cell types.

## Figures and Tables

**Table 1:** The size and zeta-potential of binary and ternary nanocomplexes.

Formulation	Size (nm)	PDI	Zeta Potential (mV)
pDNA/PEI (1:8)	626 ± 106	0.260	+12.24 ± 0.27
pDNA/PEI/HA (1:8:5)	806 ± 170	0.232	−7.49 ± 0.44
pDNA/PEI/HA (1:8:10)	649 ± 150	0.297	−12.40 ± 0.55
pDNA/PEI/HA (1:8:20)	683 ± 152	0.177	−16.05 ± 0.25

Each data value represents the mean ± S.E. (*n* = 4).
